# Anxiety and Depression in Advanced and Metastatic Lung Cancer Patients—Correlations with Performance Status and Type of Treatment

**DOI:** 10.3390/medicina60091472

**Published:** 2024-09-09

**Authors:** Roxana-Andreea Rahnea-Nita, Laura-Florentina Rebegea, Mihaela Dumitru, Radu-Iulian Mitrica, Alexandru Nechifor, Dorel Firescu, Adrian-Cornel Maier, Georgiana Bianca Constantin, Valentin-Titus Grigorean, Gabriela Rahnea-Nita

**Affiliations:** 1 Clinical Department, Faculty of Medicine, University of Medicine and Pharmacy “Carol Davila”, 050474 Bucharest, Romania; roxana.rahnea-nita@umfcd.ro (R.-A.R.-N.); radu.mitrica@umfcd.ro (R.-I.M.); valentin.grigorean@umfcd.ro (V.-T.G.); 2 Oncology-Palliative Care Department, “Sf. Luca” Chronic Diseases Hospital, 041915 Bucharest, Romania; gabriela.rahnea-nita@umfcd.ro; 3 Clinical Department, Faculty of Medicine and Pharmacy, “Dunarea de Jos” University, 800008 Galati, Romania; laura.rebegea@ugal.ro (L.-F.R.); alexandru.nechifor@ugal.ro (A.N.); dorel.firescu@ugal.ro (D.F.); adrian.maier@ugal.ro (A.-C.M.); 4 Radiotherapy Department, “Sf. Ap. Andrei” County Emergency Clinical Hospital, 800579 Galati, Romania; mihaeladumitru11@yahoo.com; 5 Radiotherapy Department, The Oncological Institute “Prof. Dr. Alexandru Trestioreanu”, 022328 Bucharest, Romania; 6 Morphological and Functional Sciences Department, Faculty of Medicine and Pharmacy, “Dunarea de Jos” University, 800008 Galati, Romania; 7 Department of General Surgery, “Bagdasar-Arseni” Clinical Emergency Hospital, 041915 Bucharest, Romania; 8 Clinical Department, Faculty of Midwifery and Nursing, University of Medicine and Pharmacy “Carol Davila”, 050474 Bucharest, Romania

**Keywords:** lung cancer, anxiety, depression, performance

## Abstract

*Background and Objectives*: The treatment of advanced and metastatic lung cancer is multimodal, and it is coordinated by a multidisciplinary team. Anxiety and depression occur frequently in patients with lung cancer, creating considerable discomfort in therapeutic management. At the same time, these psychoemotional symptoms affect the patients’ quality of life. Objective: This research seeks to identify correlations both between anxiety and depression and the patients’ performance statuses, as well as between anxiety and depression and the type of treatment: radiotherapy, chemotherapy, tyrosine kinase inhibitors (TKI), immunotherapy and palliative care. *Materials and Methods*: The study evaluated 105 patients with lung cancer from two oncologic centers. Patients were assessed for anxiety and depression using the questionnaire Hospital Anxiety and Depression Scale (HADS). The HADS is a self-report rating scale of 14 items. It measures anxiety and depression, and has two subscales. There are seven items for each subscale. There are 4-point Likert scale ranging from 0 to 3. For each subscale, the score is the sum of the seven items, ranging from 0 to 21. *Results*: The most powerful correlation with statistical significance was observed between the IT type of treatment (immunotherapy) and the normal level of anxiety, PC = 0.82 (*p* < 0.001) as well as the normal level of depression. Palliative treatment was correlated with anxiety and depression, both borderline and abnormal. For ECOG 3–4 performance status and abnormal anxiety, respectively, abnormal depression was significantly associated. Also, continuous hospitalization was associated with abnormal anxiety and depression. *Conclusions*: Early assessments of anxiety and depression are necessary in patients with advanced and metastatic lung cancer, with unfavorable performance status, who have been admitted to continuous hospitalization, and who require palliative care.

## 1. Introduction

Lung cancer is the second most common cancer in the United States, both in women and in men, and is the leading cause of cancer-related death in the United States and worldwide [1].

In the European Union, lung cancer is the most common cause of death by neoplastic disease in men and the second most common cause of death in women [2].

In Western countries, the percentages of lung cancer caused by smoking are 90% in men and 80% in women.

The specific signs and symptoms of lung cancer are cough, dyspnea, chest pain, hemoptysis, paralysis of the vocal cords, superior vena cava syndrome, Pancoast–Tobias syndrome, pleurisy and pericarditis, and the systemic symptoms are fatigue, loss of appetite and weight loss.

Treatment options in lung cancer vary depending on staging, histopathological type, molecular markers and the performance status of the patient. The treatment is multimodal, with a management that requires a multidisciplinary team and includes surgical resection, radiotherapy, chemotherapy, targeted therapy, immunotherapy and palliative therapy [3].

The majority of patients with advanced non-small-cell lung cancer are treated with immune checkpoint inhibitors and targeted therapies. These innovative treatments lead to prolonged rates of survival [4,5,6].

For advanced or extensive-stage small-cell lung cancer (SCLC), combined chemotherapy (platinum-etoposide) and immunotherapy (atezolizumab or durvalumab during and after chemotherapy) are the standards of first-line treatment, but the patients’ survival rates are modest [7].

At the same time, the early involvement of palliative care in the treatment of patients with advanced and metastatic lung cancer improves the quality of their lives. The purpose of palliative care is to improve the physical, psychological, social and spiritual symptoms of the patients, thus contributing to the improvement of treatment results.

Anxiety and depression occur frequently in patients with lung cancer, affecting their quality of life and creating considerable discomfort in therapeutic management [8,9].

Anxiety represents a feeling that appears in threatening or stressful situations [10].

Depression is a mood disorder that generates sadness and loss of interest and is the most common psychiatric condition in cancer patients [11,12].

The coexistence of psychiatric conditions and cancer has a significant impact on patients, their families and those who matter to the patient, as well as on the care team.

There is increased interest in the cumulative impacts of cancer, depression and anxiety.

Gniewko Więckiewicz et al., in a narrative review published in 2024, highlights the fact that the psychiatric conditions of cancer patients contribute to a significant socioeconomic burden for both the patients and those who care for them [12,13,14].

The Hospital Anxiety and Depression Scale (HADS) was developed by Zigmond and Snaith, and it evaluates separately the intensities of anxiety and depression (normal, borderline and abnormal) [15,16]. HADS was validated for many different patient populations, including cancer patients and HIV patients, as well as for COVID-19 survivors with long COVID [17,18,19,20]. HADS is free and available online.

The research question is: is there a relationship between psychoemotional symptoms (anxiety and depression) and the type of treatment, the Eastern Cooperative Oncology Group performance status (ECOG) and the type of hospitalization?

The justification of this research is to identify the categories of patients with lung cancer who have high levels of anxiety and depression in order to be referred to psycho-logical counseling and to be treated.

The objective of this research is to evaluate anxiety and depression in patients with lung cancer, depending on different variables: gender, age, life environment, Eastern Co-operative Oncology Group performance status (ECOG) and the type of hospitalization, as well as the type of treatment: radiotherapy, chemotherapy, tyrosine kinase inhibitors (TKI), immunotherapy and palliative care.

## 2. Materials and Methods

*Study Design*

This study evaluated patients with lung cancer from two oncologic centers: the Oncology—Palliative Care Department of the “Saint Luca” Hospital of Chronic Diseases in Bucharest and the Oncology Department of the Galati Clinical County Emergency Hospital.

All single patients with lung cancer, an ECOG Performance Status Scale of 0–4, and who were admitted in a period of 3 months (November 2023 and January–February 2024) in the Oncology—Palliative Care Department of “Saint Luca” Hospital of Chronic Diseases in Bucharest were evaluated, as well as all patients with pulmonary cancer who were admitted in a period of 2 months (January–February 2024) in the Oncology and Thoracic Surgery departments of the “Sf. Ap. Andrei” County Emergency Clinical Hospital in Galati, regardless of the date of diagnosis or the therapeutic method at the time of admission. The total number of patients included in the study was 105 patients (83 patients from Bucharest and 22 patients from Galati).

The inclusion criteria were:

- Adult patients (>18 years old) diagnosed with lung cancer in the two centers from Bucharest and Galati
- Patients who agreed to be evaluated with the HADS scale.

Exclusion criteria:

- Patients admitted and treated in the other oncologic departments of the two hospitals
- Patients who did not agree to be evaluated for anxiety and depression.

The patients were assessed for anxiety and depression using the questionnaire Hospital Anxiety and Depression Scale (HADS). The questionnaire was self-administered or administered by trained staff.

The Hospital Anxiety and Depression Scale (HADS) was developed in 1983 by Zigmond and Snaith. The HADS is a self-report rating scale with 14 items. It measures anxiety and depression and has two subscales. There are seven items for each subscale. There is a 4-point Likert scale ranging from 0 to 3. For each subscale, the score is the sum of the seven items, ranging from 0 to 21. 

The HADS questionnaire has seven items, with depression and anxiety subscales. 

Scoring for each item ranges from zero to three, where three denotes the highest anxiety or depression level. 

A total subscale score of >8 points out of a possible 21 denotes considerable symptoms of anxiety or depression.

Zigmond and Snaith have suggested two cutoff scores for detecting depression and anxiety: scores of 8 to 10 = doubtful cases and scores of 11 and higher = valid cases. Scoring: 0–7 = Normal; 8–10 = Borderline abnormal (borderline case); 11–21 = Abnormal (case) [15,21]. The role of HADS is to identify the patients who need psychological assessment and treatment.

The statements regarding anxiety were:

- I feel tense or ‘wound up.’
- I get a sort of frightened feeling as if something awful is about to happen.
- Worrying thoughts go through my mind.
- I can sit at ease and feel relaxed.
- I get a sort of frightened feeling like ‘butterflies’ in the stomach.
- I feel restless as if I have to move.
- I get sudden feelings of panic.

The statements regarding depression were as follows:

- I still enjoy the things I used to enjoy.
- I can laugh and see the funny side of things.
- I feel cheerful.
- I feel as if I am slowed down.
- I have lost interest in my appearance.
- I look forward to enjoying things.
- I can enjoy a good book or radio or TV program [15].

The time required to fill out the questionnaire was 10 min.

The ECOG performance status assesses how the disease affects the daily living abilities of the patient and determines appropriate treatment and prognosis. An ECOG performance status ranks performance status on a scale of 0 to 5. (0 = Fully active, able to carry on all pre-disease performance without restriction; 1 = Restricted in physically strenuous activity but ambulatory and able to carry out work of a light or sedentary nature; 2 = Ambulatory and capable of all self-care but unable to carry out any work activities, up and about more than 50% of waking hours; 3 = Capable of only limited self-care, confined to bed or chair more than 50% of waking hours; 4 = Completely disabled, cannot carry on any self-care, totally confined to bed or chair; 5 = Dead [22].

The ECOG Performance Status Scale circulates in the public domain and is therefore available for public use.

Approval from the Ethical Committee of “Saint Luca” Hospital of Chronic Dis-eases in Bucharest, no. 487/16.01.2023, was obtained as well as approval from the Ethical Committee of the “Sf. Ap. Andrei” County Emergency Clinical Hospital in Galati, no. 1824/28.02.2024.

The purpose of this study was explained to the patients; they freely agreed to participate in this study. Informed consent was obtained from all subjects involved in the study.

## 3. Results

We analyzed 105 patients, with a median age of 66.5 years (range: 48–86 years): 74 male patients (70.48%) and 31 female patients (29.52%). Regarding life environment, 57 patients (54.29%) were from urban areas, and 45.71% were from rural areas. Seventy-six patients (72.38%) were treated in day-care types of hospitalization, and 29 patients (27.62% of cases) were treated in continuous hospitalization. A total of 42.86% of the cases attended elementary schools, 54.29% of cases attended high schools, and 2.85% of cases went to universities. Regarding ECOG performance statuses, 45 patients (42.86%) had ECOGs = 0–2 and 60 patients (57.14%) of cases had ECOGs = 3–4. Chemotherapy was performed in 39 patients (37.5%) and immunotherapy in 23 patients (28.82% of cases) (Table 1). The levels of anxiety and depression are presented in Table 2 and Table 3.

We performed correlations between the type of treatment, the ECOG performance status and the type of hospitalization (independent variables) and the levels of anxiety and depression (dependent variables). The values of the Pearson correlation coefficient are indicated in Table 4.

The most powerful correlation with statistical significance was observed between the IT type of treatment (immunotherapy) and the normal level of anxiety, PC = 0.82 (*p* < 0.001) and the normal level of depression, PC = 0.86 (*p* < 0.001). Palliative treatment was correlated with borderline anxiety, PC = 0.32 (*p* = 0.02); borderline depression, PC = 0.45 (*p* = 0.01); abnormal anxiety, PC = 0.49 (*p* < 0.01); and abnormal depression, PC = 0.62 (*p* < 0.01) (Table 4).

An ECOG 0–2 performance status and normal anxiety were significantly associated: PC = 0.65 (*p* < 0.001). An ECOG 0–2 performance status and normal depression were significantly associated: PC = 0.52 (*p* < 0.005). 

An ECOG 3–4 performance status and abnormal anxiety were significantly associated: PC = 0.8 (*p* = 0.04). An ECOG 3–4 performance status and abnormal depression were also significantly associated: PC = 0.6 (*p* = 0.3). 

The day care type of hospitalization was associated with normal anxiety: PC = 0.78 (*p* < 0.001) and with normal depression as well: PC= 0.62 (*p* < 0.005). 

Also, continuous hospitalization was associated with abnormal anxiety and depression: PC = 0.78 (*p* = 0.04) and PC = 0.86 (*p* = 0.03), respectively (Table 4).

## 4. Discussion

This research seeks to identify correlations between anxiety and depression and the patients’ performance statuses, their types of treatment and their types of hospitalization.

The results of our study highlight the following: palliative treatment, an ECOG 3–4 performance status and continuous hospitalization are correlated with high levels of anxiety and depression, while immunotherapy, an ECOG 0–2 performance and day care are correlated with normal levels of anxiety and depression.

Individual factors (age, gender), psychological factors (non-adaptation to the disease, lack of hope, fear, change in self-image, concern for others) and social factors (education level, family, social support, income) as well as factors related to the cancer (stage of disease, prognosis, symptoms) and its treatment (radiotherapy, chemotherapy, response to treatment, adverse reactions) as well as some drugs (antiemetics, steroids) contribute to the development of depression and anxiety among cancer patients [23]. The proven link between lung cancer and smoking can cause some patients with lung cancer to blame themselves for the onset of the disease [23,24].

The results of our study highlight the fact that patients with advanced and metastatic lung cancer and unfavorable performance statuses (ECOG 3 and ECOG 4) who were admitted to continuous hospitalization and require palliative care have high levels of anxiety and depression, a fact that is explained by the lack of hope and by the factors related to cancer and its treatment.

Linden et al. [25], in a study performed during 2004–2009 on 10,153 consecutive patients at two major cancer centers, revealed different levels of anxiety and depression, depending on the cancer’s location: the highest levels of distress at the time of the cancer diagnosis were in patients with lung, gynecological or hematological cancer. This study also revealed that patients younger than 50 years and women have high levels of anxiety in more than 50% of cases [24].

A cross-sectional study performed by Zeilinger EL et al. [26] on 7509 patients with cancer showed that the prevalence of anxiety and depression was significantly higher in lung and brain cancer patients.

Naser AY et al. [9], in a study conducted on 1011 patients, which included both patients admitted to continuous hospitalization and day hospitalization, highlighted the fact that depression and anxiety have a prevalence of 23.4%, in all patients. At the same time, the prevalence was higher in cancer patients admitted to continuous hospitalization as compared to those in day hospitalization [9]. This difference is attributed to the severity of the disease or the advanced or terminal stage of the disease. This study showed that depression and anxiety have higher prevalences in patients with head and neck cancer, lung cancer and bladder cancer, as compared to other locations of cancer, as well as in patients who were admitted to continuous hospitalization [9].

Hansen MJ et al. [27], in a study carried out in Denmark during a period of 1 year (September 2016–September 2017) on 135 newly diagnosed lung cancer patients in first-line treatment (consisted of chemotherapy alone or combined with radiotherapy, immunotherapy or different combinations), highlighted the fact that patients with mild to moderate depression symptoms were less likely to complete first-line treatment. The authors of the study concluded that depression symptoms among newly referred lung cancer patients may reduce non-adherence to first-line lung cancer treatment.

Duan L. et al. [28], in a study conducted in 2022 on 302 patients with lung cancer who were undergoing chemotherapy, highlighted that the most frequent symptoms were as follows: anxiety, depression, fatigue, low physical activity and poor quality of sleep.

Prapa P. et al. [29], in a study carried out in Greece in 2020 during a period of 2 months (February–March 2020) on 135 patients with lung cancer who performed chemotherapy at a 1-day clinic, highlighted that the symptoms of the disease and the adverse effects of chemotherapy determine a low quality of life and an increase in psychoemotional stress.

Immunotherapy and targeted therapies offer longer survival, but their association with psychoemotional and physical symptoms has been less studied.

McFarland DC et al. [30], in a study conducted in 2019 in the United States of Ameri-ca on 109 patients with metastatic lung cancer, in which chemotherapy was most frequently followed by immunotherapy and targeted therapies, highlighted that immunotherapy and targeted therapies are associated with less depression as compared with chemotherapy, while the physical symptoms are the same.

Regarding emotional distress before treatment with checkpoint inhibitors, Zeng et al. [31], in a study analyzing 227 patients, found statistically significant correlations, highlighting in the analysis between emotional distress before treatment and the objective response rate significantly lower rates of response to treatment in patients with emotional distress as compared to those without it. Also, a decrease in the 2-year survival rate, 46.5% versus 64.9%, can be noted in patients with emotional distress as compared to those without it.

In our study, a correlation with statistical significance was observed between the IT type of treatment (immunotherapy) and a normal level of anxiety, PC = 0.82 (*p* < 0.001) and a normal level of depression, PC = 0.86 (*p* < 0.001). Although immunotherapy is indicated in the advanced and metastatic stages of lung cancer, the fact that patients who undergo this treatment do not experience symptoms such as anxiety and depression is explained by the fact that they have high hopes regarding the results of this treatment.

Demoralization, an existential disorder, also has a significant impact on anxiety and depression in cancer patients and it is correlated with poor quality of life [32,33,34].

The patients in this study, in whom borderline and abnormal levels of anxiety and depression were identified, were referred to psychological counseling and specialized treatment.

Limitations: The findings describe levels of emotional distress at the time of admission in the departments but cannot inform about the trajectories of anxiety and depression over time.

This research did not evaluate the link between lung cancer and the patients’ sense of guilt related to the onset of the disease.

Another limitation of the study is the absence of follow-up: patients were questioned during the treatments. Another limit might be the absence of survival analysis function of any factors (age, disease stage, performance status) or overall survival. 

The potential impact of the findings in clinical practice in the future is difficult to anticipate due to the heterogeneity of the patients and to the lower grade of patients’ compliance.

## 5. Conclusions

The most powerful correlation with statistical significance was observed between the IT type of treatment (immunotherapy) and a normal level of anxiety and a normal level of depression; palliative treatment was correlated with borderline anxiety, borderline depression, abnormal anxiety and abnormal depression.

ECOG 0–2 performance status is correlated with normal levels of anxiety and depression, while with an ECOG 3–4 performance status and abnormal anxiety, respectively, abnormal depression was significantly associated. 

Day care was associated with normal levels of anxiety and depression, while continuous hospitalization was associated with abnormal anxiety and depression.

Early assessment of anxiety and depression, treatment and monitoring during treatment are necessary in patients with advanced and metastatic lung cancer and unfavorable performance statuses (ECOG 3 and ECOG 4) who are admitted to continuous hospitalization and require palliative care.

The originality of this study resides in the analysis and correlations between the levels of depression and anxiety with the types of oncological treatment. There are only a few studies in the literature that evaluate depression and anxiety in lung cancer patients treated with immune checkpoint inhibitors.

Further studies with extensive follow-up periods of time are needed.

## Figures and Tables

**Table 1:** Patients’ characteristics.

Patients’ Characteristics	No of Patients (%), N = 105
Median age (range) (year)	66.5 (48–86)
Gender	
Male	74 (70.48)
Female	31 (29.52)
Life environment	
Urban	57 (54.29)
Rural	48 (45.71)
Type of hospitalization	
Day care	76 (72.38)
Continuous care	29 (27.62)
Studies	
Elementary school	45 (42.86)
High school	57 (54.29)
University	3 (2.85)
ECOG Performance status	
ECOG = 0–2	45 (42.86)
ECOG = 3–4	60 (57.14)
Treatment type	
Chemotherapy	39 (37.5)
Palliative treatment	23 (22.12)
Immunotherapy	30 (28.82)
Tyrosine kinase	4 (3.85)
Radiotherapy	8 (7.69)

**Table 2:** Distribution according to levels of anxiety/depression (N = 105 patients).

	Normal (N/%)	Borderline (N/%)	Abnormal (N/%)
Anxiety	60/57.14%	22/20.95%	23/21.91%
Depression	62/59.05%	17/16.19%	26/24.76%

**Table 3:** Distribution of patients according to gender, performance status ECOG, type of hospitalization, studies, life environment and levels of anxiety and depression (N = 105 patients).

	Anxiety-Normal	Anxiety-Borderline	Anxiety-Abnormal	Depression-Normal	Depression-Borderline	Depression-Abnormal
Male: 74 patients	43 (58.11%)	16 (21.62%)	15 (20.27%)	46 (62.16%)	14 (18.92%)	14 (18.92%)
Female: 31 patients	17 (54.84%)	6 (19.35%)	8 (25.81%)	16 (51.61%)	3 (9.68%)	12 (38.71%)
ECOG = 0–2 60 patients	41 (68.33%)	13 (21.67%)	6 (10%)	46 (76.67%)	7 (11.67%)	7 (11.67%)
ECOG = 3–4 45 patients	19 (45.22%)	9 (20%)	17 (37.78%)	16 (35.56%)	10 (22.22%)	19 (42.22%)
Day care: 28 patients	19 (67.86%)	5 (17.86%)	4 (14.29%)	20 (71.43%)	4 (14.29%)	4 (14.29%)
Continuous care: 77 patients	41 (53.25%)	17 (41.46%)	19 (24.68%)	42 (54.55%)	13 (16.88%)	22 (28.57%)
elementary school: 45 patients	25 (55.56%)	12 (26.67%)	8 (17.78%)	26 (57.78%)	8 (14.78%)	11 (24.44%)
high school: 57 patients	34 (59.65%)	9 (15.79%)	14 (24.56%)	3 (56.65%)	9 (15.79%)	14 (24.56%)
university: 3 patients	1 (33.33%)	1 (33.33%)	1 (33.33%)	2 (66.67%)	0 (0%)	1 (33.33%)
Urban: 58 patients	33 (56.90%)	11 (18.97%)	14 (24.14%)	32 (55.17%)	9 (15.51%)	17 (29.31%)
Rural: 47 patients	27 (57.45%)	11 (23.4%)	9 (19.15%)	30 (63.83%)	8 (17.02%)	9 (19.15%)

**Table 4:** Coefficients of determination (Pearson).

Variables	Anxiety Normal	Depression Normal	Anxiety Borderline	Depression Borderline	Anxiety Abnormal	Depression Abnormal
Type of treatment						
CMT	0.32 (*p* < 0.03)	0.16 (*p* = 0.04)	0.22 (*p* = 0.04)	0.12 (*p* = 0.4)	0.2 (*p* = 0.04)	0.4 (*p* = 0.04)
Palliative	0.12 (*p* < 0.01)	0.22 (*p* < 0.01)	0.32 (*p* = 0.02)	0.45 (*p* = 0.01)	0.49 (*p* < 0.01)	0.62 (*p* < 0.01)
RT	0.38 (*p* = 0.5)	0.52 (*p* < 0.01)	0.26 (*p* = 0.09)	0.46 (*p* = 0.89)	0.36 (*p* = 0.69)	0.16 (*p* = 0.09)
IT	0.82 (*p* < 0.001)	0.86 (*p* < 0.001)	0.39 (*p* = 0.7)	0.239 (*p* = 0.7)	0.29 (*p* = 0.6)	0.39 (*p* = 0.7)
TKI	0.3 (*p* = 0.4)	0.15 (*p* = 0.1)	0.4 (*p* = 0.08)	0.4 (*p* = 0.08)	0.4 (*p* = 0.08)	0.5 (*p* = 0.08)
ECOG performance status						
ECOG = 0–2	0.65 (*p* < 0.001)	0.52 (*p* < 0.005)	0.35 (*p* = 0.06)	0.25 (*p* = 0.06)	0.15 (*p* = 0.06)	0.18 (*p* = 0.06)
ECOG = 3–4	0.3 (*p* = 0.4)	0.1 (*p* = 0.4)	0.32 (*p* = 0.4)	0.28 (*p* = 0.4)	0.8 (*p* = 0.04)	0.6 (*p* = 0.03)
Type of hospitalization						
Day care	0.78 (*p* < 0.001)	0.62 (*p* < 0.005)	0.25 (*p* = 0.06)	0.18 (*p* = 0.06)	0.4 (*p* = 0.06)	0.3 (*p* = 0.06)
Continuos care	0.2 (*p* = 0.5)	0.3 (*p* = 0.5)	0.42 (*p* = 0.5)	0.35 (*p* = 0.4)	0.78 (*p* = 0.04)	0.86 (*p* = 0.03)

## Data Availability

Data supporting the reported results can be obtained by request to the correspondent author.
